# The Role of Osteocalcin and Alkaline Phosphatase Immunohistochemistry in Osteosarcoma Diagnosis

**DOI:** 10.1155/2018/6346409

**Published:** 2018-05-03

**Authors:** Hasrayati Agustina, Ita Asyifa, Afiati Aziz, Bethy S. Hernowo

**Affiliations:** Department of Anatomical Pathology, Faculty of Medicine, Universitas Padjadjaran/Hasan Sadikin General Hospital, Bandung, Indonesia

## Abstract

**Background:**

The diagnosis of Osteosarcoma (OSA) is not always straightforward. OSA may resemble Other Primary Bone Tumours (OPBT). The diagnosis of osteosarcoma is sometimes difficult especially in a very small specimen. Immunohistochemistry is one of ancillary testing types that can help the diagnosis of many tumours. The aim of this study was to evaluate the validity of Osteocalcin (OCN) and Alkaline Phosphatase (ALP) immunohistochemistry in discriminating OSA from OPBT.

**Method:**

This study included 50 selected human primary bone tumours, 25 cases of OSA and 25 cases of OPBT. Immunohistochemical evaluation of OCN and ALP was done for all cases. The sensitivity, specificity, positive predictive value (PPV), negative predictive value (NPV), and overall accuracy were calculated.

**Result:**

The mean age of OSA and OPBT patients was 19.6 ± 13.6 and 40.0 ± 16.3 years, respectively. Osteocalcin was positive in 17/25 (68%) cases of OSA and 16/25 (64%) cases of OPBT (*p* = 0.061). Alkaline Phosphatase was positive in 24/25 (96%) cases of OSA and 5/25 (20%) cases of OPBT (*p* < 0.001). The sensitivity of OCN in OSA diagnosis was 68%, with specificity, PPV, NPV, and overall accuracy being 36%, 52%, 53%, and 52%, respectively. The sensitivity of ALP in OSA diagnosis was 96%, with specificity, PPV, NPV, and overall accuracy being 80%, 82.7%, 95.2%, and 88%, respectively.

**Conclusion:**

ALP immunohistochemistry is useful in discriminating OSA from OPBT. ALP is superior to OCN in OSA diagnosis. OCN cannot be used to differentiate between OSA and OPBT.

## 1. Introduction

Osteosarcoma (OSA) is a malignant neoplasm in which the neoplastic cells produce bone. Osteosarcoma is the most common high-grade sarcoma of the skeleton. It has bimodal age distribution with most cases developing between the ages of 10 and 14 years and a second smaller peak in older adult (30% occur in individuals aged > 40 years) [1].

Diagnosis of OSA requires close collaboration between clinicians, radiologists, and pathologists (triple diagnosis). Although most osteosarcomas have a very characteristic radiographic appearance, there is sufficient overlap with other malignant bone tumours to make an imperative pathologic diagnosis before instituting definitive therapy [2]. In general, patients with OSA come at an advanced stage, requiring a faster time in diagnosis to improve the patient's life expectancy. Core biopsy and fine-needle aspiration are the frequent methods used to obtain the sample for pathological diagnosis. The tissue sample taken by this procedure is frequently limited resulting in difficulty in osteosarcoma diagnosis. OSA is a great histologic mimicker and poses the diagnostic challenge especially in small tissue biopsies [3]. Histopathologically and cytologically, the diagnosis of OSA relies on the presence of malignant tumour cells and neoplastic bone. Tumour cells in OSA typically demonstrate severe anaplasia and pleomorphism and may be epitheloid, plasmacytoid, fusiform, small, and round or spindled. The neoplastic bone is eosinophilic when unmineralized (osteoid) and basophilic/purple if mineralized. The osteoid matrix appears solid, homogeneous, amorphous, irregular, and lace-like or curvilinear in the background or between tumour cells. In some cases, distinguishing unmineralized matrix (osteoid) from other eosinophilic extracellular materials especially collagen may be difficult and subjective [1–4]. In order to make an accurate diagnosis, it is important to find molecular marker to distinguish OSA and OPBT.

Osteocalcin (OCN) is known to be a bone tissue-specific protein [5, 6]. Studies have shown that OCN immunohistochemistry may be helpful in distinguishing OSAs from other malignancies. OCN immunohistochemistry has been proven to be sensitive but lacks specificity [7–9].

Alkaline Phosphatase (ALP) is an ubiquitous enzyme present in all tissues but is mainly concentrated in the liver, kidney, placenta, and bone. In the musculoskeletal system, ALP is abundant in osteoblasts and is considered to play a role in the mineralization of newly formed bone [10]. In the research conducted on canine, ALP is a sensitive and specific marker in identifying tumour cells in OSA [11–13]. ALP studies on serum of OSA patients have been widely performed. Serum ALP levels correlated with patient prognosis and extent of lesions [14–16]. Research to identify the diagnostic value of ALP immunohistochemistry in human OSA cases has not been widely reported in the literature.

The present study aimed to evaluate the validity of Osteocalcin (OCN) and Alkaline Phosphatase (ALP) immunohistochemistry in discriminating OSA from OPBT.

## 2. Material and Method

This research has ethical clearance from Health Research Ethics Committee Padjadjaran University with number 419/UN6.C.10/PN/2017.

### 2.1. Patients

All study materials were paraffin blocks of patients who had surgery and had been diagnosed histopathologically as OSA and OPBT. Twenty-five cases of conventional OSA and 25 cases of OPBT (consists of angiosarcoma, chondrosarcoma, chordoma, Ewing's Sarcoma, malignant giant cell tumour, low grade fibromyxoid sarcoma, and plasmacytoma) were included in this study. All patients were from Hasan Sadikin General Hospital, Indonesia, between 1 January 2014 and 31 December 2016.

### 2.2. Histology Process and Immunohistological (IHC) Stains

All specimens were fixed in 10% buffered neutral formalin and embedded in paraffin. Tumour specimens in paraffin blocks were cut to 4 *μ*m sections, and three-section slides per specimen were prepared. One slide was stained with the standard H&E for histological evaluation by two experienced pathologists (AF and BSH). IHC techniques were performed on the remaining sections to detect expression levels of OCN and ALP. Immunohistochemistry was performed using labelled streptavidin biotin immunoperoxidase complex method with Starr Trek universal HRP detection system (Biocare Medical). Primary antibodies included were rabbit IgG polyclonal antibody for Osteocalcin (catalogue number: PB9088, Boster Biological Technology, Pleasanton, US, at 1 : 200 dilution) and rabbit polyclonal antibody to Alkaline Phosphatase (catalogue number: GTX100817, GeneTex International Corporation, Hsinchu City 300, Taiwan, at 1 : 200 dilution). The procedure used for immunohistochemistry was as follows: 4 *μ* thick sections were cut on 0.01% poly-L-lysine coated glass slides and baked at 60°C for one hour on a standard histology hotplate. Sections were dewaxed in xylene and treated with three changes of ethanol and alcohol then brought to water. Sections were subjected to heat induced antigen retrieval in a decloaking chamber (DC2008INTL, Biocare Medical, USA) in EDTA (pH 8.0), followed by cooling at room temperature for 20 minutes. Sections were then treated to block endogenous peroxidase, stained with primary antibodies, and incubated for 1 hour at room temperature. Detection was done by horseradish peroxidase polymer-based detection system (Biocare Medical) and diaminobenzidine chromogen and counterstained with haematoxylin.

### 2.3. IHC Analysis and Interpretation

Authors independently evaluated the slides and determined the mean percentage (%) of positive (PP) cells and the staining intensity (SI) in at least three different high-power fields (400x) using Olympus CX21 light microscope. Immunoreactivity for OCN and ALP was identified by the presence of cytoplasm of tumour cells and osteoid matrix. The intensity of OCN and ALP was graded semiquantitatively on a scale of 0–3 (0, no staining; 1+, weak staining; 2+, moderate staining; 3+, strong staining) and distribution of stained tumour cell was graded semiquantitatively on a scale of 0–4 (0, negative; 1+, <25%; 2+, 25–50%; 3+, 50–75%; 4+, >75%). Then, the average weighted score (AWS) for each area was calculated by multiplying PP by the SI (score 0–12) [7]. The results were score as negative (0–3) and positive (4–12)

### 2.4. Statistical Analysis

Results were tabulated with the original histologic diagnosis performed by various pathologists [1]. The comparison between OSA and ALP immunoreactivity in OSA and OPBT was evaluated using Mann–Whitney *U* test. *p* < 0.05 is considered statistically significant. The sensitivity, specificity, positive predictive value, negative predictive value, and accuracy were also calculated. Statistical tests were performed using the software SPSS 23.0 version

## 3. Results

The demographic data on cases of this study are given in Table 1. This study includes OSA and OPBT range from pediatric to adult patients. The mean age of OSA patients was lower than that of OPBT patients. The most common site of OSA in this study was in lower extremities.

### 3.1. OCN Immunoreactivity in OSA and OPBT

**Figure 1:**
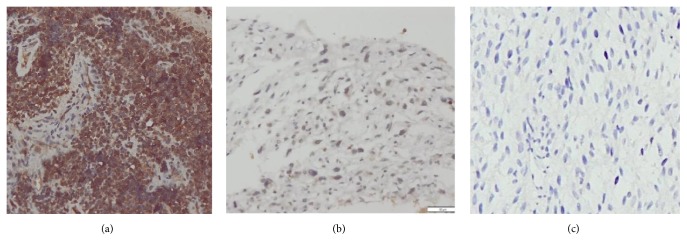
OCN immunoreactivity: (a) positive immunoreactivity of OCN in OPBT (Ewing's Sarcoma) score 12 (200x), (b) negative immunoreactivity of OCN in Osteosarcoma, score 2 (200x), and (c) negative control (Osteosarcoma, 200x).

Osteocalcin was expressed in the cytoplasm of OSA and OPBT tumour cells (Figure 1) and also on the osteoid. As shown in Table 2, osteocalcin was positive in 17/25 (68%) cases of OSA and 16/25 (64%) cases of OPBT. On applying Mann–Whitney *U* test, statistically significant result was not seen for OCN staining between OSA and OPBT (*p* = 0.061). The OCN showed sensitivity of 68%, specificity of 36%, positive predictive value of 52%, negative predictive value of 53%, and accuracy of 52% to Osteosarcoma.

### 3.2. ALP Immunoreactivity in OSA and OPBT

**Figure 2:**
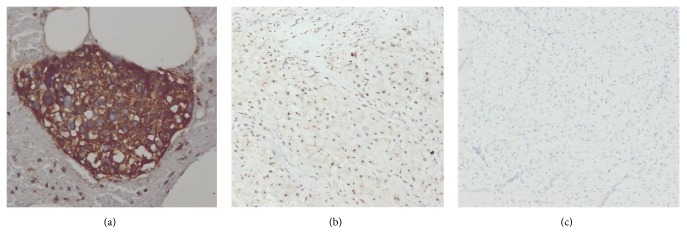
ALP immunoreactivity: (a) positive immunoreactivity of ALP in Osteosarcoma score 12 (200x), (b) negative immunoreactivity of ALP in chondrosarcoma, score 2 (100x), and (c) negative control (low grade fibromyxoid sarcoma, 100x).

Alkaline Phosphatase was expressed in the cytoplasm of tumour cells (Figure 2). Positive immunoreactivity was observed in all osteosarcomas except a fibroblastic variant, as shown in Table 2. ALP was also positive in 5/25 (20%) cases of OPBT. Positive immunoreactivity of ALP in OPBT was from chondrosarcoma cases. On applying Mann–Whitney *U* test, highly statistically significant result was obtained for ALP immunoreactivity between OSA samples and OPBT (*p* < 0.0001). The ALP immunohistochemistry showed sensitivity of 96%, specificity of 80%, positive predictive value of 82.7%, negative predictive value of 95.2%, and accuracy of 88% in diagnosing OSA.

## 4. Discussion

Osteocalcin, known as bone *γ*-carboxyglutamic acid-containing protein (BGLAP) preferentially expressed by osteoblasts, is the most abundant noncollagenous bone matrix protein and often used as a late marker for bone formation [17]. Prior to our study, Fanburg et al. studied 106 tumours immunostained with monoclonal antiosteocalcin (OCN). They included 42 OSAs, 25 non-bone-forming sarcomas, 24 other malignant tumours including lymphomas, carcinomas, and melanomas, and 15 benign bone tumours. Cytoplasmic staining with OCN showed 70% sensitivity and 100% specificity [8]. In this study, OCN immunoreactivity showed no significant difference between OSA and OPBT. The OCN showed sensitivity of 68% and specificity of 36%. Compared to that study, the sensitivity of OCN for osteosarcomas in our study was similar, but our study showed very low OCN specificity. This significant specificity difference was most probably due to the difference on the histopathological type of OPBT. In our study, the tumours selected in OPBT group were mostly sarcomas. Sarcomas that showed positive immunoreactivity for OCN in our study were 11 cases of chondrosarcoma, 3 cases of Ewing's sarcoma, chordoma, and plasmacytoma. Fanburg-Smith et al. studied 22 central nervous system and musculoskeletal mesenchymal chondrosarcomas and found that 67% of cases demonstrated acquired osteoblastic phenotype, cells positive for osteocalcin at the site of endochondral ossification [18]. Northern blot analysis has also identified OCN mRNA expressed in several nonosseous tissues [5]. These studies support the evidence that OCN was not specific for Osteosarcoma.

Compared to OCN, the diagnostic value of ALP in this study was superior. The ALP showed sensitivity of 96%, specificity of 80%, positive predictive value of 82.7%, negative predictive value of 95.2%, and accuracy of 88% in OSA diagnosis. Compared to previous ALP studies in canines, our result was equally valuable. Barger et al. examined ALP immunoreactivity of 61 cytopathology samples from canine, consisting of 33 OSAs, 4 chondrosarcomas, 4 synovial sarcomas, 2 fibrosarcoma, 9 soft tissue sarcomas, 2 plasma cell tumours, 2 lymphomas, 2 amelanotic melanomas, 2 mast cell tumours, and 1 multilobular tumour (MLT) of bone, which showed sensitivity and specificity of 100% and 89% [13]. Ryseff and Bohn examined OSA from 83 dogs, with 5-bromo-4-chloro-3-indolyl phosphate/nitroblue tetrazolium (BCIP/NBT) as a substrate for ALP and reported that the sensitivity and specificity of ALP expression detected using BCIP/NBT substrate applied to cells previously stained with Wright-Giemsa stain for OSA were 88 and 94%, respectively [11]. These studies suggested that ALP had a good diagnostic value in both human OSA and canine. In both human and canine patients, the predominant bone cancer diagnosis is OSA. OSA incidence rates in dogs are 27 times higher than in people. Utilising information from genetic studies could assist in this both species, with the higher incidence rates in dogs contributing to the dog population being a good model of human disease. Referring to our study result, there may be potential similarities that occur among molecular pathogenesis of OSA and OPBT among human and canine [19].

In conclusion, ALP immunohistochemistry is useful in discriminating OSA from OPBT. ALP is superior to OCN in OSA diagnosis. OCN cannot be used to differentiate between OSA and OPBT.

## Figures and Tables

**Table 1:** Demographic characteristics of osteosarcoma and other primary bone tumour cases of the study.

Parameter	OSA (*n* = 25)	OPBT (*n* = 25)
Age range (years)	8–60	13–63
Mean age (years)	19.6 ± 13.6	40.0 ± 16.3
Number of Males	16	15
Number of females	9	10
Male : female ratio	1.8 : 1	1.5 : 1
Site of bone tumor		
Head and neck	3	7
Trunk	0	9
Upper extremity	3	3
Lower extremity	19	6

OSA, Osteosarcoma; OPBT, Other Primary Bone Tumour.

**Table 2:** Immunoreactivity of osteocalcin and alkaline phosphatase in osteosarcoma and other primary bone tumour.

Tumor type	Total no.	OCN (*n* = 50)		ALP (*n* = 50)	
		Positive	Negative	Positive	negative
Conventional OSA	25	17	8	24	1
Chondroblastic	4	2	2	4	0
Fibroblastic	1	0	1	0	1
NOS	20	15	5	20	0
OPBT	25	16	9	5	20
Angiosarcoma	2	0	2	0	2
Chondrosarcoma	13	11	2	5	8
Chordoma	3	1	2	0	3
Ewing's sarcoma	3	3	0	0	3
Low grade fibromyxoid sarcoma	1	0	1	0	1
Malignant giant cell tumor	1	0	1	0	1
Plasmacytoma	2	1	1	0	2

*p* value		*p* = 0.061		*p* < 0.0001	

OCN, Osteocalcin; ALP, Alkaline Phosphatase; OSA, Osteosarcoma; NOS, not otherwise specified; OPBT, Other Primary Bone Tumor.
